# Effect of red sugar on functional constipation in children compared to figs syrup; a randomized controlled trial study 

**Published:** 2018

**Authors:** Pantea Tajik, Amir Hossein Goudarzian, Mahdi Shadnoush, Bahador Bagheri

**Affiliations:** 1 *Semnan University of Medical Sciences, Semnan, Iran. *; 2 *Student Research Committee, Mazandaran University of Medical Sciences, Sari, Iran.*; 3 *Department of Nutrition, Shahid Beheshti University of Medical Sciences, Tehran, Iran.*; 4 *Cancer Research Center and Department of Pharmacology, Semnan University of Medical Sciences, Semnan, Iran *

**Keywords:** Constipation, Children, Traditional medicine, Iran.

## Abstract

**Aim:**

The present study is aimed to investigate the effect of red sugar on functional constipation in children compared to figs syrup.

**Background:**

Treatment of constipation in childhood improves gastrointestinal function in the future and regular bowel habit. Red sugar is an effective ingredient in the treatment of constipation. Figs syrup is the other common natural substance used to treat constipation in children. Conducted studies on these two substances and similar herbal substances have shown their beneficial effects, but in a conducted study, it is reported that the effect of fig syrup is less than the chemical material.

**Methods:**

This Randomized Controlled Trial (RCT) Study was done in 2016. First, by performing an examination and filling out the identifying form of the patient's health status, mothers respond to the designed questionnaire. 30 children with constipation were treated with the usual drug, fig syrup, and 30 other children received red sugar. After a 4-week treatment period, the examination was conducted again and the questionnaire was filled out again. The changes following the intervention were measured and the status before and after treatment were compared as well. The analyses were performed using SPSS 20 (SPSS for Windows, SPSS Inc., Chicago, IL, USA).

**Results:**

In this study, there was no significant difference between effects of red sugar and fig syrup in terms of the frequency of fecal excretion, and pain at the time of excretion (p = 0.264). However, the fig syrup was more effective in reducing the anorexia (p < 0.001) and abdominal pain compared with fig syrup (p < 0.001). Also fig syrup was more effective in inducing diarrhea (p = 0.019).

**Conclusion:**

In general, treatment by red sugar has been effective in improving the functional characteristics of constipation in children; and did not show any complication and toxic effects. It is easily accessible at affordable prices to resolve childhood constipation.

## Introduction

 According to released statistics, constipation is a common gastrointestinal problem in children and adults, which impose a lot of expense to the society (1). Despite its high prevalence and its benign nature, constipation causes great stress and anxiety in families (2). In fact, constipation is the second leading cause for referring to pediatric gastroenterologists. Researchers estimate that the incidence of constipation in hospitalized children is up to 43% (3). About 3% of the complaint of outpatient visits of pediatric gastroenterologists and 10-25% of children's digestive system visits are regarding this problem. It seems that functional constipation is the main cause of constipation in children (about 95% of the causes of constipation) and has a widespread outbreak (4, 5). 

According to the findings of the studies, 90 to 95% of constipations in children are not associated with any disorders (6). Causes of constipation in children include hypothyroidism, celiac disease, false intestine obstruction in the context of neuropathic and myopathic diseases; but in general, it can be stated that constipation is commonly associated with several causes that lead to disorders in various levels of the natural process of fecal excretion (7). Constipation during childhood causes abdominal and intestinal pains, as well as mood diseases such as body heat rise and moisture overload, burns and abscesses (8). Many of the babies' troubles and confusion with unknown causes are due to constipation and gastrointestinal problems (5). Treatment of constipation in childhood improves gastrointestinal function in the future and regular bowel habit (9). 

 At present, there are not any uncomplicated and effective therapies for the relief of this problem, especially in children with a specific sensitivity (10). According to the results of other studies and systematic reviews on treatment of constipation in children, it can be concluded that there are not any strong evidence proving that laxative treatments provided better results compared with placebo (11); until now, polyethylene glycol has been shown more effective results in treatment of constipation (12). In general, common constipation treatments are associated with some problems, while there are many drugs in Iranian traditional medicine books for the treatment of constipation (13). Using traditional experiences increases the likelihood of discovering effective drug substances; and reduces the cost of producing medications (14). Therefore, new strategies are aimed at shortening processes to reduce costs and to facilitate accessing the effective drugs. One of these strategies is using medicinal plants and drugs used in traditional medicine for centuries. As a result, reviewing measures to expand non-edible treatments is important in treating constipation (15).

 The selected product in this study is Red Sugar, which, according to studies, is an effective ingredient in the treatment of constipation (16). Brown sugar or red sugar is a sugar cane sugar, which is red or brown in color clearly. The red color is attributed to the presence of sugar syrup (molasses) (17). Figs syrup is the other common natural substance used to treat constipation in children. Conducted studies on these two substances and similar herbal substances have shown their beneficial effects, but in a conducted study, it is reported that the effect of fig syrup is less than the chemical material (18).

 Therefore, more precise studies are needed to be conducted to investigate the effect of these herbal substances on constipation of children and to compare their effects with each other. Therefore, the present study is aimed to determine the effect of red sugar on functional constipation in children compared to figs syrup. 

## Methods

This Randomized Controlled Trial (RCT) Study was done in 2016 (June-December). The statistical population included all children aged 2 to 10 years old who referred to the clinic of Amir Al momenin Hospital in Semnan for suffering from functional constipation. According to the prevalence of the disease and previous studies, the sample size was estimated to include 60 children.

**Sampling method**

**Diagram 1:**
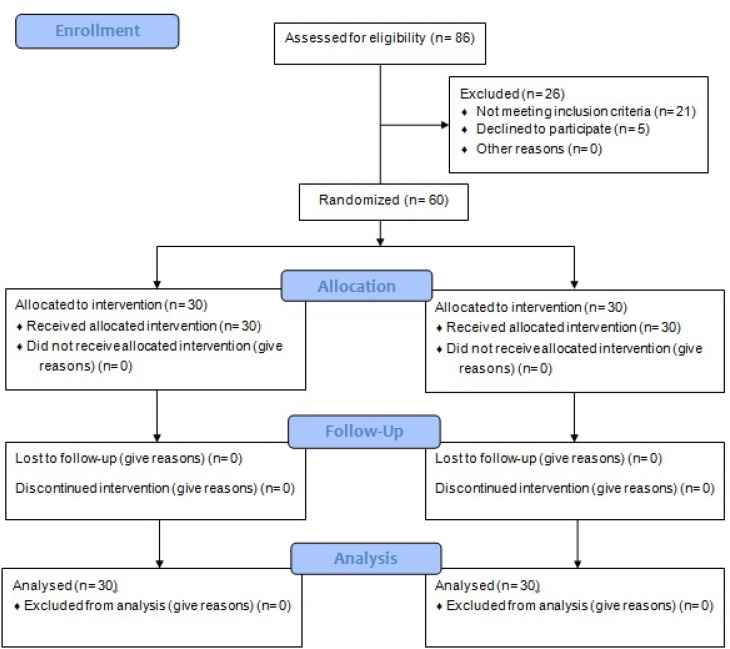
The CONSORT Flow Diagram of the sampling stages

 A total of 60 eligible patients suffering from functional constipation referred to the clinic of Amir Al momenin Hospital in Semnan, Iran were investigated. Due to the prevalence of congenital and Hirschsprung diseases, newborns under the age of 2 years, and childrens with hypothyroidism (based on medical profile) were excluded from the study. Moreover, the causes of constipation were investigated and children with non-functional constipation were excluded from the study by a pediatrician (based on diagram 1). Participants were randomly divided into two 30 member groups (experimental and control). At first, 60 random numbers between 0 and 1 were made by computer (Excel software), and then, numbers were printed automatically. An envelope containing a number was given to each subject. Children with numbers less than 0.5 were assigned into the experimental group and the ones with the numbers more than 0.5 were assigned into the control group.

**Table 1:** Comparison the effect of red sugar and fig syrup on constipation of children

	Red sugar		Fig syrup		*p*
	Before, n (%)	After, n (%)	Before, n (%)	After, n (%)	
Fecal excretion					0.264
* less than 2 times a week*	5 (16.6)	0	7 (23.3)	0	
* 2-3 times a week*	15 (50)	0	12 (40)	0	
* More than 3 times a week*	10 (33.4)	30 (100)	11 (36.7)	30 (100)	
* p*	<0.001		<0.001		
Pain					0.315
* Yes*	18 (60)	0	25 (83.3)	0	
* No*	12 (40)	30 (100)	5 (16.7)	30 (100)	
* p*	<0.001		<0.001		
Fecal incontinence					0.003
* Yes*	3 (10)	1 (3.3)	5 (16.7)	0	
* No*	27 (90)	29 (96.7)	25 (83.3)	30 (100)	
* p*	0.009		0.005		
Anorexia					<0.001
* Yes*	25 (83.3)	3 (10)	23 (76.6)	10 (33.3)	
* No*	5 (16.7)	27 (90)	7 (23.4)	20 (66.7)	
* p*	<0.001		<0.001		
Abdominal pain					<0.001
* Yes*	10 (33.3)	0	12 (40)	9 (30)	
* No*	20 (66.7)	30 (100)	18 (60)	21 (70)	
* p*	<0.001		0.009		
Diarrhea					0.019
* Yes*	0	14 (46.6)	5 (16.7)	24 (80)	
* No*	30 (100)	16 (53.4)	25 (83.3)	6 (20)	
* p*	0.007		<0.001		

**The method of implementing the plan**

 This study was performed in Amir Al-Momenin Hospital in Semnan and the special clinic of Semnan University of Medical Sciences. At first, physicians performed the examination. Then by collaboration of parents, a questionnaire regarding the child's condition, including the rate of growth, frequency of excretion, stool diameter, fecal consistency (high, medium, or low), having pain at fecal excretion (high, medium, or low), appetite status, fecal incontinence, secretion of mucous material from the anus and being in fecal retention status was filled out (Justification of constipation in children can be made due to the frequency of fecal excretion and change in this habit, that is, a decrease in the frequency of fecal excretion). Half of the children with constipation were treated by common known methods by fig syrup and the other half were treated by red sugar for one month. These two materials based on reports had no significant side effects (19). The amount of prescribed product varies according to the weight of the baby, and its criterion for each drug was based on 2gr / kg / day and 2cc / kg / day. After calculating the prescribed dose according to the weight of the child, the prescribed medicine was provided.

 The determined amount of red sugar powder was dissolved in a cup of hot water and consumed. Two weeks after the treatment, the initial assessment including the efficacy and possible side effects of the medication was done; and if the results were positive, the treatment continues for another 2 weeks. Totally, the duration of the treatment lasted up to one month; and after passing the mentioned period, the patient's biography was examined. The questionnaire was reevaluated after completion of the treatment period and the results were compared to the primary results.

**Data analysis**

 Data analysis was performed using SPSS 20 (SPSS for Windows, SPSS Inc., Chicago, IL, USA). Basic descriptive for quantitative variables was presented using mean (SD) and n (%) for qualitative variables. Chi-square test, two way ANOVA, independent T-test and paired T-test were used as inferential statistical tests. Statistical significance was set at *P*< 0.05.

**Ethical considerations**

 This study was conducted in accordance to the conventions laid out within the Helsinki Statement (20) and was approved by ethics committee of Semnan University of Medical Sciences (IR.SEMUMS.REC.1394.17). Also this study was approved in Iranian Registry of Clinical Trials (IRCT2015061722794N1). Parents of children were (1) informed about the study's aims and procedures, (2) informed about the voluntary nature of their participation, and (3) given assurances that any/all interventions would not affect their conventional medical care before signing an informed consent document. Parents of children confidentiality was assured by completing all of the study procedures in a quiet treatment area. All personal data were anonymized by using codes when referring to participants. 

## Results

60 children aged 2 to 10 years including 40 boys and 20 girls, 30 of which received red sugar powder and the other 30 were treated using fijan figs containing figs and senna extract. In each group, 20 boys and 10 girls with the same age and functional constipation symptoms were placed.

 Based on results shown in table 1, four weeks after consuming red sugar powder, 20 patients had convenient daily fecal excretion while 10 children had one day in between excretion and also in fig syrup group, 18 children had daily excrements while 12 had one day in between (*p* = 0.264). Both substances had effects on reducing the pain during fecal excretion and no significant differences were observed (*p* = 0.315). 

Red sugar was more effective in reducing the anorexia (*p* < 0.001) and abdominal pain compared with fig syrup (*p* < 0.001). In terms of diarrhea, after treatment, in red sugar group no patient had diarrhea and in the fig syrup group, 5 (16.66%) children suffered diarrhea. After treatment, there was a significant difference between the two groups; since fig syrup can make the stool more watery and causes diarrhea due to the presence of the Sena (*p* = 0.019).

## Discussion

In this study, red sugar had similar effect with fig syrup in the treatment of constipation, and after treatment with red sugar, no side effects were observed during the two months of treatment. In general, based on the results of this study, there were no significant difference in the frequency of fecal excretion, stool diameter, and pain during excretion between red sugar and fig syrup groups. However, fig syrup was more effective in treating fecal incontinence. Red sugar was more effective in reducing anorexia and relieving pain during excretion.

 In a study conducted by Levine (21) on 100 children with constipation and fecal incontinence among the patients treated with edrolex, 50 cases with fecal incontinence were cured. Of the 50 patients treated with the Senate, all patients with fecal incontinence were cured; and there was no statistical significant difference between these two groups. In the study of Taitz, Wales (22), conducted on 250 children with constipation problems, about 100 patients experienced pain during fecal excretion, 50 patients suffered abdominal pain during excretion and 30 patients suffered fecal incontinence. All patients suffered functional constipation with thick stool diameter. Patients were classified into two groups. One group was treated by plants including fig and the other group was treated with Pedrolex. Eventually, 125 patients treated with piodrolex 100% responded to treatment; and all symptoms of the patients had improved. However, in 125 people who were under herbal treatments, including figs, 25 did not respond to treatment and still had constipation symptoms. In a study done by Tabbers, DiLorenzo (23) investigated children under the age of 18 with constipation. They found that physical activity is effective in the treatment of constipation. Taking probiotic routinely is not recommended. In this study, the use of piodelux was recommended for evacuation of the intestine and stool mass, and the use of senna was prohibited in the long term and chronic form. In 2009, van den Berg, Dijkgraaf (24) investigated 90 children with functional constipation; 45 children were treated with oral piodelux and 45 were treated with piodelux enema. Treatment of 80% of children in the enema group and 60% of the children in oral pyridoxine group were successful. It was recommended that in cases of chronic constipation, piodelux enema should be used firstly so that the stool mass easily to be excreted.

 In traditional medicine book, red sugar has been mentioned for treating the constipation; that as a laxative agent can stimulate bowel movements and improve fecal excretion. Given that red sugar can increase appetite, it is used to treat constipation and anorexia (25, 26). In the present study, in the red sugar group, 25 patients (83.33%) suffered from anorexia at the beginning; after consumption of red sugar, the appetite improved in all patients. In figs syrup group, 23 (76.66%) patients suffered from anorexia at the beginning; flowing the treatment, anorexia still remained in 10 (33.33%) patients. There was a significant difference between the two groups; and the red sugar was very effective on improving appetite and anorexia. In addition, studying on the therapeutic effect of red sugar and fig syrup on the functional constipation showed no side effects during treatment with red sugar and patients were satisfied with treatment with red sugar. In total, red sugar as a traditional medicine can be used for treatment of childhood functional constipation.

**Limitations**

 This study’s most important limitations are that only volunteers participated and there were few children who suffered from severe symptoms. Moreover, we had difficulty contacting a larger sample.

**Implications for Nursing Practice**

 It is very important to know the effects of figs syrup and red sugar on constipation of children for nurses to prepare holistic plans for symptom management. Symptom management training for children with constipation should include the use of red sugar. Furthermore, periodically repeated symptom management training will reduce symptom frequency. During such training, whether at home or in the hospital, symptoms should be well defined and knowledge related to symptom management and assessment of education programs should be provided to these children.

**Implications for Researchers**

 This research suggests that the effects of figs syrup and red sugar on constipation of children should be studied with more details.

 In conclusion, red sugar has been effective in improving the functional characteristics of constipation in children; and has no toxic effects. Besides, it is easily accessible at affordable prices to help resolve childhood constipation problem. It is recommended that children with functional constipation use red sugar as an uncomplicated herbal remedy.

## Conflict of interests

The authors declare that they have no conflict of interest.
